# Survival After Discharge From Geriatrics vs. Internal Medicine Wards, by Risk Status and Diagnosis

**DOI:** 10.1093/geroni/igaa057.458

**Published:** 2020-12-16

**Authors:** Antonella Giordano, Giulia Carreras, Mauro Di Bari, Luca Degli Esposti, Paola Michelozzi, Roberto Bernabei, Niccolò Marchionni, Daniela Balzi

**Affiliations:** 1 University of Florence, Florence, Italy; 2 Clicon – Health, Economics & Outcome Research, Ravenna, Italy; 3 Regione Lazio, Roma, Italy; 4 Fondazione Gemelli, Rome, Italy; 5 University of Florence, Firenze, Italy; 6 ASL Toscana Centro, Florence, Italy

## Abstract

In randomized clinical trials, compared to Internal Medicine (IM), admission to Geriatrics (G) improved clinical outcomes of frail older patients accessing the Emergency Department (ED). Whether this advantage is maintained also in the “real world” is uncertain. We compared long-term survival of patients admitted to G or IM wards after stratification for background risk and across a variety of discharge diagnoses. Data were derived from the “Silver Code National Project (SCNP)”, an observational study of 180,079 unselected 75+ years old persons, admitted via the ED to IM (n=169,717, 94.2%) or G (n=10,362) wards in Italy. The Dynamic Silver Code (DSC), based on administrative data, was applied to balance for background risk between participants admitted to G or IM. One-year mortality was 33.7%, it was lower in participants discharged from G than IM (32.1 and 33.8%, respectively; p<0.001), and increased progressively across four DSC risk classes (p<0.001). Admission to G was associated with survival benefit in DSC class II to IV participants, with HR (95% CI) of 0.88 (0.83-0.94), 0.86 (0.80-0.92) and 0.92 (0.86-0.97), respectively. Cerebrovascular diseases, cognitive disorders, and heart failure were the ICD-9 coded diagnoses with the widest survival benefit from admission to G, which was mostly observed in DSC class III. In conclusion, admission to G may provide long-term survival benefit in subjects who, based on the DSC, may be considered at an intermediate risk. Specific clinical conditions should be considered in the ED to improve selection of patients to be targeted for G admission.

